# Digital Twins’ Advancements and Applications in Healthcare, Towards Precision Medicine

**DOI:** 10.3390/jpm14111101

**Published:** 2024-11-11

**Authors:** Konstantinos Papachristou, Paraskevi F. Katsakiori, Panagiotis Papadimitroulas, Lidia Strigari, George C. Kagadis

**Affiliations:** 1 3dmi Research Group, Department of Medical Physics, School of Medicine, University of Patras, 26504 Rion, Greece; up1111465@upatras.gr (K.P.); vkatsak@upatras.gr (P.F.K.); panospap@upatras.gr (P.P.); 2 Bioemission Technology Solutions, BIOEMTECH, 15344 Athens, Greece; 3 Department of Medical Physics, IRCCS Azienda Ospedaliero-Universitaria di Bologna, 40138 Bologna, Italy; lidia.strigari@aosp.bo.it; 4 Department of Imaging Physics, The University of Texas MD Anderson Cancer Center, Houston, TX 77030, USA

**Keywords:** healthcare, digital twins, precision medicine, artificial intelligence, personalized medicine

## Abstract

This review examines the significant influence of Digital Twins (DTs) and their variant, Digital Human Twins (DHTs), on the healthcare field. DTs represent virtual replicas that encapsulate both medical and physiological characteristics—such as tissues, organs, and biokinetic data—of patients. These virtual models facilitate a deeper understanding of disease progression and enhance the customization and optimization of treatment plans by modeling complex interactions between genetic factors and environmental influences. By establishing dynamic, bidirectional connections between the DTs of physical objects and their digital counterparts, these technologies enable real-time data exchange, thereby transforming electronic health records. Leveraging the increasing availability of extensive historical datasets from clinical trials and real-world sources, AI models can now generate comprehensive predictions of future health outcomes for specific patients in the form of AI-generated DTs. Such models can also offer insights into potential diagnoses, disease progression, and treatment responses. This remarkable progression in healthcare paves the way for precision medicine and personalized health, allowing for high-level individualized medical interventions and therapies. However, the integration of DTs into healthcare faces several challenges, including data security, accessibility, bias, and quality. Addressing these obstacles is crucial to realizing the full potential of DHTs, heralding a new era of personalized, precise, and accurate medicine.

## 1. Introduction

Digital Twins (DTs) are dynamic, virtual representations of physical systems, objects, or processes that are continuously updated with real-time data from their physical counterparts. These models integrate sensor data, advanced algorithms, and machine learning to simulate, monitor, and predict the behavior of the physical entity they represent. The DT concept has transformed several fields and industries due to its ability to provide a virtual model of physical entities or systems, enabling real-time study, monitoring, and optimization of performance and operations [1,2]. By evolving based on incoming data, DTs offer precise analysis and optimization, facilitating informed decision-making across diverse applications. As automation and digitization are showing an increasing trend and Industry 4.0 is arriving, DTs are gaining popularity, ‘exploding’ the interest in applications extending from industrial and engineering enterprises to healthcare [3,4]. In healthcare, DTs represent a pioneering approach that aims to achieve a complete digital representation of patients, termed Digital Human Twins (DHTs), and attempts to enhance disease prevention (prognosis), diagnosis, and treatment [5]. They can provide proper models of various aspects of the human body and therefore facilitate drug interaction research, treatment effectiveness, and personalized medicine. By illuminating complex operations of physical systems, DTs provide the potential of in silico simulations, thus easing precise predictions of systems’ responses under diverse conditions [6]. This is a promising and developing approach that helps in adapting disease treatments and preventive interventions to the unique characteristics of each individual patient, thus making DTs a natural and complementary strategy [3].

DT application entails a complex framework comprising diverse skills, professional expertise, and technological resources [6]. This framework encompasses large-scale biological data repositories, cloud computing infrastructures, standardized protocols for data acquisition and communication, multidisciplinary collaborations, artificial intelligence (AI) algorithms, virtual reality (VR) technologies, and robust legal and ethical frameworks. Their basic model consists of many categories but there are three key components, i.e., the physical product, its physical representation, and the connection between these two, allowing data and information flow [7]. Marr et al. claimed that DTs can take advantage of contemporary technologies like AI, data analytics, and smart sensors, to dynamically bridge the physical and digital domains, improve system performance, and explore novel opportunities [8]. DT technology has much to gain from the rise of the Internet of Things (IoT) as it can combine real-world data from diverse sources and facilitate comprehensive simulations of physical entities and their functionality through time [3].

As health and lifestyle management tools and services become more widely available, the need for higher-quality care is increasing [9,10,11]. To enhance medical diagnostics, prognoses, treatments, and overall patient well-being, the use of DTs is currently being investigated in dynamic human models and simulations.

The current review presents the application of the growing and promising DT technology in the medical environment as well as the consequences and challenges of its use in personalized medicine and public health.

## 2. Methods

**Figure 1:**
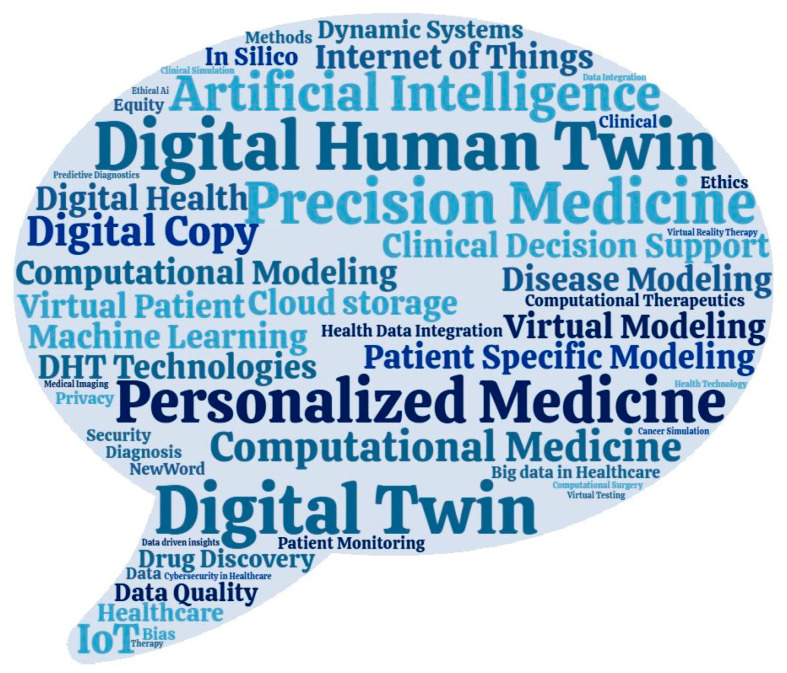
A word cloud produced by the keywords of the papers reviewed in this study.

This review study was based on a comprehensive search conducted across multiple databases, including PubMed, IEEE Xplore, and Scopus. We utilized relevant keywords such as ‘Digital Human Twin’, ‘precision medicine’, ‘IoT’, ‘AI’, cloud computing’, and ‘virtual reality’ to identify studies focused on Digital Human Twin (DHTs) and their applications in healthcare (Figure 1). The research was restricted to studies from the last decade to capture the most recent advancements. Initially, 168 papers were identified [12].

Following the initial check, we screened titles and abstracts, removed duplicates, and excluded papers that did not align with DHT technologies or precision medicine. Articles were further filtered using specific inclusion criteria, prioritizing peer-reviewed studies published over the last ten years. Selected articles needed to discuss technologies like the Internet of Things, cloud storage and computing, artificial intelligence, and virtual reality, or their applications in precision medicine, such as person-centered risk stratification, rapid diagnosis, disease modeling, surgical planning, targeted therapies, and drug discovery.

After this filtering process, we refined our selection to 74 articles that met the inclusion criteria. These articles were classified into three major themes. The first theme, DHT technologies, was further divided into subcategories such as IoT, cloud storage and computing, AI, virtual reality, and data standards. The second theme, precision medicine, organized studies into areas like person-centered risk stratification, rapid diagnosis, disease modeling, surgical planning, targeted therapies, and drug discovery. The third theme, issues and considerations, focused on challenges surrounding data quality, bias, security, privacy, equity, and accessibility.

Each of the 74 selected articles underwent a thorough review to extract key findings, insights, and implications. This synthesis informed the discussion and conclusions of this paper, providing a comprehensive understanding of the role of the Digital Human Twin in precision medicine, contextualized within the latest advancements in the field.

**Figure 2:**
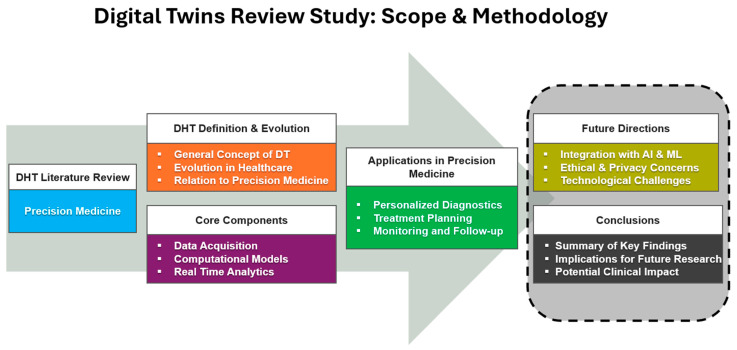
Methodology and structure of the review study on Digital Twins in healthcare applications.

The overall methodology and structure of this are depicted in Figure 2.

## 3. The Digital Human Twin (DHT)

DHTs stand as one of the latest developments in healthcare technology and are based on a smooth, two-way data exchange between virtual and physical entities. This exchange allows for targeted interventions based on predictive simulations and real-time updates to the digital model. Cellina et al. state that the objective of DHTs is to generate digital human representations that capture their physiological, anatomical, and ideally cognitive characteristics. However, to fully utilize DHTs, advanced analytics and visualization tools that can process biological data spanning multiple scales [6]. Furthermore, additional data can be incorporated into DTs, such as demographics, social habits, and questionnaires, and data can be derived from the digital health records of the patients (drugs, pathologies, treatment with ionizing radiation, etc.). To better understand the difference between DTs and DHTs, Digital Twins are virtual models of physical systems used to simulate, monitor, and optimize non-human entities, such as industrial machines or processes. In contrast, Digital Human Twins are a specialized form of DTs focused on replicating human physiology for healthcare applications. DHTs leverage patient-specific data to simulate biological systems, enabling personalized medical interventions. For example, DHTs can predict how a patient’s body will respond to a specific drug, improving treatment precision. Since this review refers to DT applications in the field of medicine, DHTs will be used henceforth.

In general, DHTs can present several forms like the full body, organ- or tissue-specific systems, and even cellular and molecular models. They can also be customized to represent specific diseases or circumstances allowing for personalized interventions and therapies. DHT instances, which are exact replicas of a DHT, provide in silico testing and comparisons to determine the most effective course of treatment for specific patients [3]. Although several companies and research teams are currently investigating the creation of digital replicas for specific body parts or physiological systems, the development of a complete DHT remains a future quest [5].

Shengli et al. [13] notes that digital replicas of people are essentially presented on their accounts on social media. These social media copies illustrate people’s thoughts, feelings, and other aspects of their lives. Similarly, DHTs are a digital representation of a human that includes their social, mental, physical, and biological characteristics [13]. As a result, their role is to develop a digital copy of a person’s life that can help in self-care, self-reflection, and personal development. Although it should clearly be noted that social media offers a complementary perspective on behavioral and emotional states, it should not be considered a primary source for constructing DHTs in a clinical context. Instead, such data may be supplementary [14] and should be validated with more reliable sources like medical records, physical assessments, and genetic data to build a comprehensive and accurate Digital Twin of a patient.

Undoubtedly, DHTs are becoming increasingly popular in the medical field. By entirely changing the medical field, they deliver live, personal health data sources for healthcare-based learning systems to foretell the possible risks specifically for older people and chronic diseases. Digital Twin instances of the same person may be used for in silico testing and comparison of different treatments or preventive or late effect mitigation intervention approaches to explore the optimum option for that individual.

To construct a valid and precise DHT, extensive healthcare data repositories are needed. Data must be characterized by high quality, accuracy, and completeness, and should include genetic data, medical records, imaging, histopathology, and other appropriate sources [13]. The setup of the dataset/platform demands that the information be standardized and harmonized, through the development and refinement of a specific ontology [15].

These data are subsequently processed by advanced analytic and AI techniques like deep learning models to improve the sophistication of DHT simulations and provide predictive decision support systems (DSSs) [5,16,17,18,19,20,21,22,23,24]. To this end, multiple professionals such as physicians, oncologists, radiation oncologists, nuclear medicine physicians, radiologists, medical physicists, geneticists, molecular biologists, psychologists, bioinformaticians, computer scientists, data scientists, and engineers should collaborate interdisciplinarily to implement DHTs and create reliable and precise digital replicas [25].

## 4. DHT Technologies

**Figure 3:**
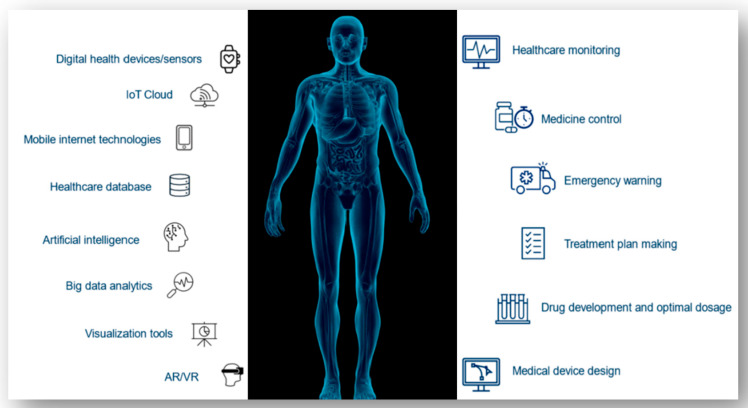
DHT technologies: working scheme.

Various technologies are currently being used in the development of DHTs (Figure 3). Digital health sensors and devices gather information directly from the patient and/or surroundings, and afterwards, they transmit and store it in real time in the IoT cloud. The construction of a Digital Twin of a patient at different stages of a disease is made possible by big data analytics and AI, which extract meaningful information from massive amounts of data [26,27]. To this end, access to IoT, cloud computing, AI, simulation, visualization tools, and machine learning models are deemed necessary to create DHTs. Developments in these and other fields of technology, like supercomputers and VR, are making the application of DHTs in healthcare more and more appealing.

### 4.1. Internet of Things

Improved IoT sensors and devices are internet-connected sensors and devices that can be embedded in everyday objects or be directly worn on the body such as wearables. These objects can gather, transmit, and receive information about the physical object and/or its surroundings. Sensor technologies and wireless networks further advance DHT applications by increasing the amount and accuracy of collected real-world and real-time data as well as sharing capabilities. The end point is a better ability to create and maintain more comprehensive simulations of physical twins, their functionality, and the changes they go through over time [3].

### 4.2. Cloud Storage and Computing

DHTs necessitate substantial data volumes and significant computational power to enable clinicians to extract real-time patient information. However, the data storage and processing demands often surpass the available resources within existing healthcare infrastructures. Furthermore, as patient data are classified as sensitive health information, there are potential risks associated with data breaches or unauthorized access, which could lead to privacy violations or even severe consequences for patients in cases of cyber-attacks (see Section 5.3). To address these challenges, many healthcare institutions outsource their data management and monitoring tasks to robust cloud-based servers. The computational burden of DHTs on healthcare facilities can be alleviated by integrating DHTs with cloud storage and cloud-based computing services. DHTs facilitate the creation of digital representations of patients, allowing for continuous health monitoring, while cloud servers provide efficient data storage and query services. To enhance data security, healthcare providers commonly employ encryption techniques before transferring data to the cloud [28,29].

### 4.3. Artificial Intelligence

DHT applications provide a real-time digital representation of the physical object by utilizing software analytics, machine learning, and AI technologies. Based on compiled data and medical histories, the DHTs of AI-based human biological systems or organs currently assist in diagnosing present medical conditions and forecasting potential future health issues [30]. Furthermore, AI helps with the design of DHTs by using physiological data from the organs to produce a 3D image. Siemens Healthineers has already used a vast database with more than 250 million annotated images, reports, and operational data to create a DHT model. This AI-based DHT model allows for digital heart design using patient data under the same conditions (size, ejection fraction, muscle contraction, etc.) [2].

### 4.4. Virtual Reality

DHTs could be combined with VR technology to create lifelike simulations. In other words, medical educators can practice treatments and procedures on virtual patients before applying them to actual ones. As a result, clinicians can practice difficult procedures and medical education can be improved [31]. For instance, pediatric cardiologists at Stanford University’s Lucile Packard Children’s Hospital have already employed VR technology as a teaching aid to clarify complex congenital heart defects by displaying a generic model of a 3D beating heart [32].

### 4.5. Data Standards

Incorporating healthcare data standards such as HL7, FHIR, and the OMOP Common Data Model is crucial for the effective implementation of DHTs. These standards facilitate the interoperability, sharing, and integration of health data from various sources, enabling the creation of accurate and comprehensive digital representations of patients.

HL7 (Health Level Seven): This set of international standards is designed to facilitate the exchange of healthcare information. It provides frameworks and standards for the integration, sharing, and retrieval of electronic health information, which is essential for the effective functioning of DHTs [28].

FHIR (Fast Healthcare Interoperability Resources): Developed by HL7, FHIR is a modern standard that utilizes web technologies to promote interoperability in healthcare data exchange. It allows for easier integration of various health applications, making it easier to implement DHTs that require real-time data from multiple sources.

OMOP Common Data Model (CDM): This model provides a standardized structure for health data, facilitating the analysis and sharing of data across different healthcare systems. By employing OMOP CDM, DHTs can efficiently process large volumes of data, enabling better insights into patient health [28,33].

## 5. Precision Medicine

Precision medicine, also known as ‘personalized medicine’, stands as an emerging approach to disease treatment and prevention. Its goal is to provide the right treatment to the right people at the right time and thus involves the use of new diagnostics and therapeutics tailored to patients’ specific genetic, biomarker, phenotypic, physical, or psychosocial characteristics [2]. Precision medicine values individual variability and, consequently, puts aside the current ‘one-size-fits-all’ treatments [34]. As a result, the effectiveness and efficiency of the healthcare system can be ameliorated.

One of the greatest obstacles to precision medicine is that patients with the same disease do not respond effectively to the same treatment. In modern healthcare, diagnosis frequently depends on an increasing but still relatively small number of biomarkers with limited sensitivity or specificity [35,36]. In addition, the complexity of the focal condition can involve altered interactions between thousands of genes that differ in patients with the same diagnosis, identifying multiple diseases hidden behind the same diagnosis. For these reasons, in most healthcare systems, personalized treatment is not possible, especially in diseases (such as cancer) with multi-stage diagnosis and treatment processes as well as high variability in disease characteristics [37].

The application of DHTs can enhance the development of precision medicine as it can assist in developing a human model that consists of the entirety of an individual’s structural, physical, biological, and historical traits. This model could then be compared with thousands or even millions of similar data points from other people, thus enhancing the identification of interesting genetic characteristics. By considering the personal history of real twins as well as the current circumstances, including time, place, and activity, DHTs may be able to predict an illness more accurately [2,34]. Moreover, DHTs can help physicians and other medical professionals like hospital pharmacists make decisions by simulating the effects of a treatment on these patients. In this review study, the following individual- and group-specific use cases have been identified and discussed:

Individual Use Cases:

i. Personalized risk models for disease prediction;
ii. Knee health monitoring through Personalized Digital Twins (PDTs);
iii. Personalized care for type 1 diabetes;
iv. Personalized cancer follow-up care;
v. Individual-simulated surgery.

Group-Specific Use Cases:

i. Customized treatment systems for European citizens;
ii. Precision cardiology with Cardiac Digital Twins (CDTs);
iii. Chronic disease management in groups like type 2 diabetes patients;
iv. Multiple sclerosis management;
v. Drug discovery for specific groups (non-Hodgkin’s lymphoma).

### 5.1. Person-Centered Risk Stratification and Prevention

Kukushkin et al. described that DHTs could influence disease risk stratification by combining data from numerous sources, including healthcare records, medical interviews, and wearable technology. They offer an organized framework for controlling this huge amount of data, enabling the creation of modified screening plans for earlier diagnosis and improved prognosis [38]. Genetic factors, medical images, and social determinants are just a few of the many patient data points that DHTs can include. The role of environment, behavior, and genetics in the pathogenesis of disease is clarified by this integration, especially regarding lifestyle and occupational diseases [5]. In addition, DHTs help healthcare professionals optimize population-level screening procedures, which promotes individualized healthcare and improves patient outcomes [28]. They also enable the predictable evaluation of screening programs and treatments, allowing the formulation of specific programs for high-risk people or groups [27,39]. DHTs can predict the beginning or development of diseases by evaluating data from numerous sources, such as wearables and electronic health records, enabling preventive therapy. Suzuki et al. illustrated the potential of DHTs in customized medicine by applying computational fluid dynamics to create a tailored risk model for minor intracranial aneurysm ruptures [40].

Lehrach et al. presented a customized treatment and disease prevention system for European citizens which involves the use of ‘virtual twins’ or ‘guardian angels’ [28]. These models simulate patient biology in Europe throughout a range of disease states using data from multiple sources, including imaging, clinical, and sensor data. As big data and computer technology become more widely available at reasonable rates, physicians will be able to create personalized digital models to test therapies before implying them to patients. Supporting this project would lower healthcare expenses while raising living standards throughout Europe.

Knee abnormalities, including osteoarthritis, congenital problems, and traumas, are common in all age groups and the primary source of disability. With the complicated structure and function of the knee, Personalized DTs (PDTs) hold great potential for monitoring knee health. PDTs enhance the delivery of individualized care by giving medical personnel deeper insights into the diseases of patients through the collection and analysis of knee data. In addition to expanding knowledge of knee health, the suggested PDT-based framework allows physicians and patients to work cooperatively to make decisions that are personalized for rehabilitation and value-driven for knee care. This framework, which is especially useful for osteoporosis prevention, combines cutting-edge technologies such as blockchain, IoT, DHTs, and AI to give smart, individualized solutions [41].

A substantial field of research relates to the development of precision cardiology via the application of Cardiac DTs (CDTs) [34]. These CDTs aim to shift from mere descriptions to predictive capabilities regarding a wide variety of conditions. They are anticipated to improve the integration between anatomical, mechanistic, and functional insights into the cardiovascular system and sophisticated analytical models built around pertinent data. The business and academic communities have expressed interest in this topic. A commercial utilization of DT technology that provides adjustable electrical and muscular characteristics was introduced in 2015. It was designed to mimic the function of the human heart. This software, called ‘Living Heart’, could turn a two-dimensional (2D) scan of a person into a detailed model of their heart, allowing users to interact with the virtual heart model [42]. A recent study that focused on both model accuracy and computational efficiency during the construction process outlined a workflow for expediting the generation of CDTs [43,44].

### 5.2. Rapid Diagnosis

Through data analysis, DHTs can identify patients at risk for developing chronic diseases. DHT-based machine learning algorithms are nowadays utilized to comprehend patterns and make predictions, and likewise assist in the early diagnosis of several conditions (cancer, asthma, diabetes, heart disease, multiple sclerosis, etc.) [45,46]. According to Rohit Kaul et al., AI combined with DHTs utilizing data gathered from a variety of sources will further support clinicians in making decisions about cancer diagnosis, prognosis, and treatment options. Compared to other decision support systems, the combination of AI and DHTs offers a deeper and more applicable illustration of data, information, and knowledge that is relevant to them in real time [47]. DHT technology has already been applied in the prediction of diseases through physical examination, the reduction in errors in diagnostic devices [4], the diagnosis and treatment of migraine [48], and stroke diagnosis. The utilization of DHTs in treatment plans allows for the examination of various approaches and the prediction of medication reactions and prognosis.

### 5.3. Disease Modeling and Clinical Decision Support

To enable immediate intervention or appropriate treatment modification, DHTs can be used to implement an alerting system in the event of critical abnormalities, unexpected changes or lack of treatment-beneficial effects. This appears to be especially appropriate in complicated chronic diseases (like multiple sclerosis) with a variable course and various potential treatments [27]. Despite the development of some organ replicas that may be used for remote monitoring of organ functionality and for testing new treatments prior to their actual use on patients, DHT systems are still primarily utilized in research and have not seen much use in daily clinical practice [5].

According to Baillargeon et al., a 3D cardiac map can be created simultaneously through the combination of electrophysiological ECG signals and morphological computed tomography scan data (ECVUETM, CardioInsight Inc., Cleveland, OH, USA) [49]. The goal is to generate heart models that produce a spherical virtual representation of a patient’s heart functionality including blood flow dynamics, contracting mechanics, and electrical impulses [49]. Similar systems could be used to predict the effectiveness of treatment, better characterize the disease, plan interventional procedures, and simulate the effects of a drug on cardiac dynamics and the effects of a pacemaker prior to actual implantation [5]. Physicians can be informed when a dangerous arrhythmia occurs by using similar maps to continuously monitor a patient’s heart activity remotely [50]. An illustrative case involves elderly individuals with type 2 diabetes, who require ongoing glucose monitoring and a personalized medication plan to prevent hypo- and hyperglycemia in the short term and long-term complications [51].

### 5.4. Surgical Planning

DHTs can provide a significant alternative approach to surgical planning, patient care, and postoperative monitoring. While DHTs have the potential to offer real-time guidance during surgical procedures through detailed visualizations and precise lesion localization [1,52], their current applications are mainly in preoperative planning and simulations. As highlighted by Cromeens et al. [53], DHTs aid surgeons in evaluating incision sites and predicting potential challenges during surgery, such as postoperative portal hypertension. However, real-time updates during surgery rely on future developments in real-time data acquisition and AI-generated alerts rather than constant physician monitoring.

Furthermore, combining DHTs with augmented data or virtual reality technologies offers surgeons the chance to create an immersive and interactive planning environment. As a result, they can more easily and better understand the anatomy of the patient, as well as achieve more organic surgical planning and decision-making processes. An example is presented by Irmici et al., focused on brain tumor surgery, as neurosurgeons can plan precise surgical interventions while preserving the patient’s quality of life thanks to DHTs which closely mimic the morpho-functional organization of cortico-subcortical circuits [54].

The complicity of DHTs could also signify a new era in organ transplantation procedures across various stages, from pre-transplant planning to post-transplant monitoring [55]. Combining machine learning and deep learning algorithms, they use historical data from various cases to predict the progress of transplantation and anticipate potential complications such as organ rejection or even failure. Previous studies have demonstrated that DHTs are capable of creating virtual replicas by integrating recipient anatomical data, such as CT or MRI scans, which help surgeons plan surgical procedures, assess transplant feasibility, and identify obstacles. They further assist with donor–recipient matching by evaluating variables like tissue compatibility and blood type. They also provide early detection of anomalies and complications in the post-transplant phase by continuously updating with real-time data from the transplanted organ and comparing it with predicted behavior. Moreover, they also make it easier to customize implants or prostheses based on a patient’s particular anatomy, which enhances fit and functionality and, in the end, improves patient outcomes and lowers the risk of complications.

Moreover, DHTs can simulate individual surgical operations. By considering the unique conditions of specific patients, individualized simulated surgery using Personalized Digital Twins (PDTs) helps prevent potential dangers and determines the most suitable devices and techniques for surgical procedures.

### 5.5. Targeted Therapies

Many diseases in the field of medicine present significant challenges because of their complex and multifactorial nature, often linking complex molecular pathways and environmental factors. This complexity develops barriers for physicians in accurately predicting the efficacy of therapies, based exclusively on clinical data. The introduction of personalized or targeted therapies into clinical practice is still a challenging prospective, even if numerous researchers highlight their promising potential. On the contrary, a huge part of research in oncology indicates that targeted therapies present a critical path in improving patient survival in most diseases.

The rise of DHTs is about to lessen the gap between clinical trials and real-world patient care. DHTs use advanced virtual computational methods to capture each patient’s unique genetic, metabolic, and environmental characteristics. As a result, they give the opportunity to choose highly personalized and efficient therapies that focus on the molecular mechanisms characterizing the disease in every single patient, by digitally representing these factors. This personalized approach has the potential to completely change the way diseases are faced and shift the medical paradigm toward one that is more patient-centered.

Disease progression and treatment response can be altered with the provision of unique tumor imaging signatures and molecular landscapes by DHTs, leading the way to the creation of innovative therapeutic strategies. Utilizing DHTs, physicians may be able to more easily simulate the effects of treatments and predict the results of their work, allowing them to improve current treatments and create new, patient-specific interventions [3,27].

Moving from theory to practice, DHTs present satisfying results across many important medical cases. Iacobucci et al. demonstrated DHTs in the management of type 1 diabetes, in which DHTs adjusted insulin dosages based on continuous blood glucose monitoring, leading to optimized glycemic control [56]. In a similar way, but now in the case of multiple sclerosis, they enabled the development of personalized therapeutic pathways by analyzing thorough disease parameters and patient characteristics [27].

Voigt et al. and Walsh et al. are also interested in the treatment of multiple sclerosis, a chronic multidimensional disease, that is known to be a common cause of neurological disability in young people [27,57]. Established research indicated that this disease is characterized by enormous complexity and heterogeneity, leading to vast amounts of data needing to be collected, given the chance for data-driven approaches. DHTs can develop diverse genomic prototypes of patients that would assist in assessing the effects of various intervention strategies and therapies. Digital Twins can be used to generate a variety of genomic prototypes of patients, which can help in evaluating the impact of different intervention strategies and therapies. Additionally, modeling parameters that are not related to genetic traits but are relevant to the patient—like environmental factors, side effects from treatment, and costs associated with managing the disease—can assist the patient by allowing predictions about how the disease will progress or how the treatment will affect them [27,57].

Cho et al. used Digital Twins to provide appropriate orthodontic treatment to Korean adult females by carefully assessing their facial profiles with facial scans and three-dimensional (3D) imaging (cone-beam computed tomography) [58]. To account for the differences in facial structures between Korean and Caucasian patients, the authors created a 3D DHT of each patient under study by combining their facial scan and CBCT (cone-beam computed tomography) image to provide more accurate measurements and assessments of the ‘sagittal relationship’ between the maxillary central incisors and forehead.

Follow-up care after cancer treatment is essential and vital for all survivors, involving routine medical check-ups to monitor their health and distinguish any potential issues created by the treatment. DHTs present a promising way to collect extensive biomarker data—such as blood tests, heart rates, activity levels, sleep patterns, and other diagnostic data—from cancer survivors. Combining these data with historical electronic medical records (EMRs), a personalized framework based on DHTs may develop individual health profiles and compare them with the corresponding healthy ones. Physicians can then evaluate the possibility of cancer recurrence and its treatability based on this analysis. Relying on a patient’s specific health conditions, they can also determine adapted surgical interventions. Finally, taking advantage of the capabilities provided by AI and simulation software, doctors can investigate patient-specific datasets to find various treatment choices and recommend the most suitable personalized care and therapy for each cancer patient [37].

### 5.6. Drug Discovery and Development

DHTs are being used more and more by the biopharmaceutical industry for a variety of purposes, most notably drug development and discovery [59,60]. According to Subramanian et al. [61], the process of developing a Digital Twin specifically for the liver entails incorporating information about diseases, disorders, and drug effects into an ordinary differential equation mathematical framework. The impacts of medication treatment, disease progression, and normal function are all accurately modeled by this virtual liver. This liver twin in combination with experimental measurements provides important new information about drug-induced liver injury [61].

Recently, Susilo et al. [62] created a virtual model focusing on non-Hodgkin’s lymphoma to support a phase I study of the antibody medication monsunetuzumab. They examine possible treatment biomarkers and dose–response relationships using DHTs. To predict effective doses and differentiate between responders and non-responders, virtual patients are generated using real patient data as well as in vitro/in vivo data and multiple DHTs are created. Tumor size, rate of proliferation, and initial T-cell infiltration are important variables that affect the extent to which a medication is effective. Characterizing disease-related environmental factors and controlling the amount of data needed for trustworthy DHTs are still difficult tasks though [62].

Key studies summarizing the main findings in healthcare are presented in Table 1.

### 5.7. Case Studies Deployment of DHTs in Healthcare

#### 5.7.1. Personalized Treatment in Oncology

A promising application of Digital Twins in oncology lies in personalizing cancer treatment. In 2020, Björnsson et al. reported the use of Digital Twins to predict treatment responses for individual patients, allowing for tailored therapies in cancer care. This study demonstrated that patient-specific models could simulate various treatment responses, enabling oncologists to select the most effective approach for each patient. Such advancements showcase the potential for improved patient outcomes and minimized side effects by optimizing treatment based on simulations [63].

#### 5.7.2. Surgical Planning and Optimization

Digital Twins have shown practical value in preoperative planning for high-risk cardiovascular surgeries. In 2020, Corral-Acero et al. detailed a study where Digital Twins modeled patient hearts, allowing surgeons to simulate interventions and anticipate complications before surgery. This approach contributed to a reduction in postoperative complications by approximately 15% and enabled personalized surgical plans, improving recovery times and patient outcomes. These findings illustrate DTs’ impact on precision in surgical interventions, with tangible benefits in cardiovascular care [34].

#### 5.7.3. Chronic Disease Management with IoT-Enabled Digital Twins

In managing chronic diseases such as diabetes, IoT-enabled Digital Twins provide a real-time monitoring solution that informs individualized care plans. In 2024, Katsoulakis et al. reported how these platforms can track vitals like glucose levels continuously, allowing clinicians to adjust treatment dynamically. This continuous feedback loop, implemented in pilot studies, improved glycemic control in patients and reduced the need for hospitalization, emphasizing DTs’ role in enhancing patient quality of life through precision health management [44].

#### 5.7.4. Predicting Outcomes in Cardiovascular Disease

Digital Twins are increasingly used to predict outcomes for patients with cardiovascular disease. The 2020 study by Corral-Acero et al. on heart failure patients demonstrated that Digital Twin models could forecast patient responses to medications, aiding in the personalization of treatment plans. This predictive capability resulted in a 25% improvement in outcomes compared to standard care, underscoring DTs’ potential in advancing precision medicine by informing more targeted and effective therapies [34].

## 6. Issues and Considerations

### 6.1. Data Quality

Medical data gathered via health scans, imaging systems, and blood tests are affected by hospital data collection procedures as well as the quality of these data. For instance, excellent image quality is rarely achieved in computerized tomography (CT) scans of heart patients, and the results typically depend on the radiology staff’s skill; this is mostly obvious in the case of less-experienced staff. Although sensors can effectively gather data and transfer them to DHTs, hospital data collection procedures remain costly and time-consuming [30].

Experts predict that the next significant developments in DHTs will attempt to resolve issues like noisy, small-scale health data rather than advances in AI research [34]. DHTs require precise and thorough data from multiple sources such as wearables, medical devices, and electronic health records. However, it can be quite difficult to guarantee the accuracy, consistency, and interoperability of these various datasets. Imprecise or untrustworthy DHT models can arise from inconsistent or lacking data [64,65].

Modern AI/machine learning and new knowledge discovery depend heavily on data—especially high-quality data. Not just ‘big data’ but data of all sizes are essential. For best results, more conventional “small data(sets)” should also be carefully chosen, categorized, and used—alone or in conjunction with “big data”—where and when appropriate. An appropriately sized dataset should be chosen over the “biggest” data in each situation. To prevent a “data swamp” and subpar, untrustworthy results, it will be essential to implement standardized metadata, pertinent ontologies, including controlled clinical terminologies, and other tried-and-true best practices in (Digital Twin) data management.

### 6.2. Data Bias

A balanced dataset that allows the comparison of any individual’s data is necessary for the creation of DHTs. Nonetheless, racial, gender, and other demographic sources of bias (e.g., white men are more represented) are currently present in many datasets related to healthcare. For any patient who does not match the dataset’s typical demographic profile, using these datasets to create human DHTs without any correction would amplify the bias that already exists and lead to a recommendation system that is not ideal [65]. Therefore, multicenter and multi-institutional data should be utilized to incorporate information from diverse sources, ensuring that digital health technologies are not biased toward a single institution’s patient population. Furthermore, it is essential for algorithms to be equitable to prevent exacerbating existing healthcare disparities [66].

### 6.3. Security and Privacy

Extensive patient data collection and storage are necessary for the effective implementation of DHTs in the healthcare industry. Confidentiality and security are crucial, but the access to and integration of such sensitive data—including biological, physical, and lifestyle data—by insurance companies or healthcare organizations raise ethical questions [34]. To prevent unauthorized access, security breaches, and potential misuse of these sensitive data, it is essential to establish strong data privacy and security measures.

Insurance companies, for example, might use newly processed data points (such as eating habits and physical activity) through DHTs to precisely differentiate premiums. This clearly constitutes a misuse of DHTs and is especially concerning for those whose health data point to an upcoming negative event that could prevent them from receiving care at a crucial time [65]. In addition, continuing concerns regarding the security of DHT databases highlight the indisputable danger of cyber-attacks [64]. To deal with these problems, anonymization or at least pseudo-anonymization of the patient data should be ensured.

To secure people’s private health information, addressing these issues necessitates stringent compliance with data protection laws, such as the Health Insurance Portability and Accountability Act (HIPAA) and the General Data Protection Regulation (GDPR) [67,68,69,70]. Furthermore, following well-established security guidelines such as ISO/IEC 27001 is essential for reducing the risks related to unapproved data collection [71].

Despite the presence of regulatory frameworks and security guidelines (such as ISO/IEC 27001), these are part of a longer-term effort to safeguard patient data and ensure proper integration across systems. At present, there are no immediate fixes to fully address the risks posed by the large-scale collection, storage, and use of DHT data. While pseudonymization and encryption offer layers of protection, they do not entirely mitigate the risks, and advancements in secure cloud technologies and improved regulatory protocols are necessary.

### 6.4. Equity and Accessibility

DHTs can likely behave as a ‘social equalizer’ providing significant benefits in society such as elevated precision in public health interventions. However, obstacles still exist in terms of accessibility, especially in personalized healthcare. The ‘digital divide’ may be worsened by the fact that not all people or communities have access to DHT technology [72]. The development and implementation of DHT must strictly follow the accessibility and equity principles to lessen these problems. To stop more healthcare disparities, it is imperative to guarantee that everyone has access to this technology, regardless of socioeconomic status, race, or location. Availability, affordability, algorithmic biases, and ethical considerations must be evaluated to ensure fair and equitable distribution [73].

### 6.5. Further Considerations

For safety-critical Digital Twin (DT) systems, several essential criteria must be met, including robustness, accountability, transparency, and fairness. Robust DTs should withstand changes in data inputs, maintaining consistent performance. Accountable DHTs are dependable in critical situations and capable of reviewing past forecasts and deductions, which ties closely to transparency. Fairness is crucial, ensuring that DT outputs treat all representative subsets, such as user subpopulations, equitably. Transparency is key to identifying points of failure through data inputs and model predictions, and it allows for accurate and high-quality interpretation of model behavior. This can be achieved through interactive descriptions of model behavior or by quantifying predictive performance using techniques such as out-of-distribution analysis, traditional performance metrics (e.g., cross-validation), and uncertainty quantification (UQ) [74].

Once data are collected for DT systems, several steps are necessary to prepare them for use. Data harmonization ensures consistency across multiple sources by standardizing formats, references, and units. Multicenter data collection requires adherence to common guidelines and protocols to maintain uniformity. Privacy and data security are critical, with strong measures needed to protect sensitive information, while data anonymization is essential for safeguarding participant privacy by removing or modifying identifiable information. Feature extraction focuses on identifying key features or biomarkers, such as segmenting imaging or biological data. Finally, the creation of structured databases ensures that processed data can be easily stored, retrieved, managed, and analyzed, enabling the DT system to function effectively.

## 7. Discussion and Conclusions

DHTs have the potential to revolutionize healthcare by providing a comprehensive view of an individual’s health data, including genetic information, medical history, and habits. This wealth of information forms the foundation for personalized treatment plans and could drive the development of tailored screening programs for early diagnostics. By identifying patterns that suggest disease presence, predicting probabilities, and tracking progression, DHTs are poised to transform the future of healthcare, particularly in the realm of personalized treatments and interventions. Moreover, with advanced features such as digital thread tracing and tracking, DHTs enhance transparency and the interpretability of AI-driven healthcare solutions. Their vast databases may also prove invaluable for clinical trial matching and other medical applications. However, these capabilities bring significant ethical concerns, particularly regarding informed consent, data ownership, and the potential for discrimination based on health profiles.

Current literature highlights DHTs’ ability to integrate diverse health data, which supports personalized care, early diagnostics, and AI-driven clinical decision-making. Strengths include comprehensive data aggregation, advances in AI, and enhanced patient engagement, making DHTs valuable for personalized treatments and predictive healthcare. However, significant challenges remain. Key concerns include data privacy, security risks, and the lack of standardized regulations around consent and data ownership. Bias in AI models is also a concern, potentially leading to inequitable outcomes, particularly for underrepresented populations.

Future research should focus on creating robust data governance frameworks, ensuring AI transparency and fairness, and developing secure data-handling protocols to ensure the ethical use of DHTs. Addressing these gaps is crucial for the broader adoption and success of DHTs in healthcare ensuring the safeguarding of patient privacy while maximizing the potential benefits of these transformative technologies.

## Figures and Tables

**Table 1:** Table of applications on key reference studies summarizing the main findings in healthcare.

Reference	Field of Application	Type of Digital Twin	Technology Used	Main Findings
Abramovici et al. (2017) [1]	Product Reconfiguration	Product Twin	IoT, Data Analytics	Enables real-time product reconfiguration during use phase, improving adaptability and efficiency.
Barricelli et al. (2019) [2]	General Applications	General Digital Twin	Survey Analysis	Provides comprehensive DT definitions, applications, and design insights for various fields.
Kamel Boulos & Zhang (2021) [3]	Precision Public Health	Human Digital Twin	Data Integration, Health Monitoring	Explores DTs’ potential to improve public health through personalized interventions.
Sun et al. (2022) [4]	Healthcare	Healthcare Twin	AI, Real-time Monitoring	Examines DTs as a transformative tool for predictive and personalized healthcare.
Armeni et al. (2022) [5]	Evidence-Based Medicine	Healthcare Twin	Data Analytics, System Modeling	Argues for DTs in evidence-based medicine to improve patient outcomes and decision-making.
Cellina et al. (2023) [6]	Personalized Medicine	Human Digital Twin	Data Analytics, Health Data Integration	Highlights DTs’ role in precision medicine, particularly in tailoring treatments.
Grieves (2019) [7]	Manufacturing Systems	Product/Process Twin	Simulation, Virtual Modeling	Discusses DTs’ applications for manufacturing, emphasizing virtual-physical integration.
Liu & Lin (2024) [10]	Construction	Construction Process Twin	Digital Modeling, IoT	Details DTs’ applications in steel construction, enhancing project efficiency and risk management.
Corral-Acero et al. (2020) [34]	Precision Cardiology	Cardiac Digital Twin	Biomechanical Modeling, Data Fusion	Presents a DT framework for personalized cardiology, enabling tailored interventions.
Wickramasinghe et al. (2022) [37]	Cancer Care	Personalized Cancer Twin	AI, Predictive Modeling	Proposes a DT framework to support personalized treatment planning in oncology.

## Data Availability

Not applicable.
